# On the Distribution of the Crinoidea in the Western States

**Published:** 1852-07

**Authors:** L. P. Yandell


*On the Distribution of the Crinoidea in the Western States.* By Prof. L. P. Yandell.—Having been engaged for several years past with my friend, Dr. B. F. Shumard, in collecting materials for a monograph on the crinoidea of the Western States, I propose to submit a few details respecting the distribution of the remains of this interesting class of animals through the palæozoic rocks, of this portion of the Mississippi valley. The richness of this quarter of our country in these fossils has already been made known to the scientific world by the labors of the late lamented Dr. Troost, whose monograph on the crinoidea of Tennessee is in course of publication under the auspices of the Smithsonian Institution. The list of the great naturalist of Tennessee includes about thirty genera and a hundred species, most of which, when he wrote, were undescribed.

The collection of Dr. Shumard and myself includes specimens from Ohio, Indiana, Illinois, Iowa, Missouri, Tennessee, Kentucky, and Alabama, extending from the lower Silurian rocks to the beginning of the coal series, and amounts to about twice the number of species described by Dr. Troost. In the present state of our knowledge we would not venture to pronounce positively on the number, but we believe it will be found rather to exceed than fall short of thirty-five genera and two hundred species, of which a few are described by Prof. Hall, in the Palæontology of New York, some have been published by Drs. Owen and Shumard, in the Journal of the Academy of Natural Sciences of Philadelphia, and many are embraced in the forthcoming work of Dr. Troost ; but a great part are new.

One of the first facts which arrested our attention in regard to our crinoids, is the limited geographical range of all but a few species. While we met with the same brachiopods and corals over wide areas, we soon had occasion to remark that encrinites were limited to a few narrow localities. *Caryocrinus ornatus* is perhaps the most widely distributed of our species. It is found in Louisville, Ky., and in Decatur county, Tenn., as well as at Lockport, N. Y., where it was first discovered. One of Dr. Troost's new species is also widely diffused—the *Agaricocrinus tuberosus*—having been found at White's Creek Springs, near Nashville, Tenn., in the vicinity of Scottsville, Ky., and at Warsaw, Ill.; and the same is true of *Olivanites verneuili*, Troost (formerly *Pentremites verneuili*), which occurs abundantly in the upper strata of the Falls of the Ohio, and presents itself in the States of Ohio and Indiana wherever those strata are exposed. A

few other species occur at various points, but much the greater number appear to have been restricted to a single spot.

With so circumscribed a range, it will not be thought singular that nearly all the species in our cabinets are peculiar to the Mississippi valley. I am aware how unsafe it is to decide hastily as to the identity or diversity of species bearing a close resemblance to each other, but after the full investigation I have had time to give the subject, my impression is, that not a single one yet found in the Western States is common to Europe and this country. Drs. Owen and Shumard incline to the belief that *Platycrinus granulatus*, and *Poteriocrinus plicatus* belong to our formations. It may be so, but if the European and American species are identical they form solitary exceptions amid a great multitude of forms. We have traced analogy everywhere, but examples of identity are still doubtful. The analogy between our species and those of Europe occurring in equivalent systems is, indeed, one of the most striking and interesting facts discovered in the progress of our researches. The *Cystidea* of Russia, Sweden, and England have their analogues in the Silurian rocks of Kentucky and Tennessee. Troost's *Echinocrinus fenestratus* answers to species of Echino-encrinus which occur in Russia; and we have lately discovered in the neighborhood of Louisville, and in Decatur co., Tenn., specimens of what Prof. Hall pronounces a *Spheronites*, corresponding to the same form which has been found in the Silurian strata of England. *Caryocrinus*, which is peculiarly American, is represented by *Hemicosmites* at St. Petersburg. The *Agaricocrinus tuberosus* of Troost has a striking resemblance to *Actinocrinus amphora*, found in equivalent formations in Ireland. The genera *Platycrinus*, *Actinocrinus*, and *Eucalyptocrinus* occur in the rocks of our country which are equivalent to those affording them in Europe, and contain species which it is difficult to distinguish from their European analogues—so difficult, indeed, that several have been taken for the same.

We have had many opportunities to remark the exceedingly limited vertical range of our crinoidea, as other observers had done before, and are prepared to assert with great confidence, that not one of our species occurs in more than one formation. For the most part, each is contained in a thin and well-defined stratum, beyond the borders of which it is seldom to be traced. Not a single species is common to the lower and upper Silurian systems, or to the Silurian and Devonian, or to the Devonian and Carboniferous, much less to the three systems; and the same, with slight qualifications, might be said of genera, as will presently appear. No fossils, it is believed, will be found to be more characteristic of their containing strata, or to mark more precisely the limits of succeeding systems, than the crinoidea.

We have met with crinoidal remains at a point lower in the geological scale than they were ever before discovered. Dr. Shumard first saw their broken columns in Wisconsin and Minnesota, several hundred feet below the magnesian limestone, in strata corresponding to the Potsdam sandstone of New York—showing that this curious race of beings were among the earliest tenants of the deep. They appeared with the primeval brachiopods and crustaceans, and have maintained their existence through every successive epoch, but in varying proportions, and in singularly diversified forms. In the oldest Palæozoic rocks we detect only, scattered here and there, the fragments of their stems and bodies. In the Trenton group of New York, and in its equivalent rocks in the West, we find a few genera and species, in a good state of preservation. In the Niagara group, at the middle of the Silurian system, we discover a sudden and great expansion of the race, which soon after seems to have begun to decline, and to have been less abundant throughout the Devonian era. But in the age which followed, they attained a great development; the Carboniferous era was the age of the crinoids. Thus, in our collections, we have from the lower Silurian system six genera and six species; and from the upper beds of that system, chiefly the Niagara group, we have ten genera and forty species. From the Devonian rocks, we have ten genera and seventeen species; while from the Carboniferous system we have twelve genera and one hundred and fifty species.

I do not suppose that these figures represent accurately the ratio of the species in the several systems, for our opportunities for collecting from all have not been equally good; but I feel assured that they will be found to be an approximation to the truth. At Louisville, the Devonian rocks are well displayed; but we have found them less rich in these fossils than the strata of an earlier age; and from one or two localities in the Carboniferous formations, we have obtained a greater number of species than this system has hitherto afforded. The fact seems to be a general one, that the crinoidea experienced a remarkable increase about the middle of the Silurian epoch—that they were stationary, if they did not decline, during the Devonian era, and reached their utmost expansion in the ages that succeeded. It has been remarked by Professor Hall, as true of the formations of New York; and the same has been the experience of European geologists.

With the succession of formations, we have a remarkable shifting of genera, and change of form, and external ornament. *Glyptocrinus*, *Schizocrinus*, *Heterocrinus* and *Agelacrinu* cease with the older Silurian strata, and are succeeded by *Haplocrinus*, *Eucalyptocrinus*, and by *Caryocrinus*, *Balanocrinus*, *Cupellæcrinus*, and many undetermined genera peculiar to our continent. These, in turn, are left behind when we ascend to the Devonian series, in which we find other forms and new genera. Here we meet with a crinoid with four bulky arms, destitute of a stem—a free-moving encrinite, showing thus early an approach in this family towards the starfishes, placed just above them in the animal scale. At this point, too, occurs our first *Actinocrinus*, which, like the forms abounding in the preceding series, is remarkable for the beauty of its external ornament. And here we also encounter a new crinoidal type—the *Pentremites*—already prefigured in *Eucalyptocrinus* of the middle Silurian period. Dr. Troost described as *Pentremites Reinwardti*, a form which he was the first to discover in Decatur county, Tenn., and which, from its associated fossils, has been generally assigned to the Silurian age. Strictly speaking, *Pentremites Reinwardti*, though bearing the closest analogy to that group, is not a pentremite, but must be placed in a new genus along with some other organisms found in rocks of a similar age. From the fact that it is found in Tennessee accompanied by *Calceola*, a Devonian fossil, and that we also find it at the Falls of the Ohio, in strata unquestionably Devonian, I do not hesitate to refer it to this system. Along with it, at the latter locality, two or three species occur closely related to it, together with *Olivanites Ver-*

*neuili* Troost, and one or two species of the same genus. Just above the beds containing these forms, the limestone series cease, and the black slate is superimposed. With the incoming of this formation, we lose all vestiges of the crinoids, until we reach an intercalated stratum of limestone near its summit, where encrinital remains again become abundant. This stratum of encrinital limestone is regarded as the base of the Carboniferous system, containing as it does among its imbedded fossils, *Orthis Michelini*, *O. crenistriæ*, *Terebratula Roissyi*, *Spirifer striatus*, *Productus punctatus*, *P. semireticulatus*, *Phillipsia*, *Griffithidis*, and encrinites of forms known only to that system in Europe.

The aspect of the crinoids of this period is eminently distinctive—in some respects denoting inferiority to the older forms, in others indicating advancement. The exquisitely ornamented surface has, for the most part, disappeared, and a plain exterior succeeded; but with it we have tentaculated fingers, and a generally more complicated organization. In this age *Actinocrinus* attained its full development, and the species of *Cyathocrinus*, if they did not appear here for the first time, only became abundant at this period. *Platycrinus*, incorrectly placed in the older series, had its beginning with this formation, and was accompanied by *Dichocrinus* and *Synbathocrinus*, by Troost's *Conocrinus*, *Agaricocrinus*, *Catillocrinus*, and by many other new, undetermined genera. The graceful *Agassizocrinus* of Troost, a stemless, free-swimming form, like the asterias, was rife in this age, and had for its companions *Melonites* and the true *Pentremites*, which latter must have swarmed in the seas that gave origin to our mountain limestone. *Pentremites florealis* occurs near the base of this formation, and is found in all its divisions up to the topmost strata. With the pentremites, we find still another novel form, intermediate between them and the new genus *Melonites*—the *Granatocrinus cidariformis* of Troost; and here, too, we meet with *Cidaris* and other echinoderms.

This succession of genera is a striking feature in the history of the crinoidea, and their extinction at various periods, affords a problem of great interest to the zoölogist. Why they should, one after another, have perished from the early seas in which they lived, to be succeeded by other and still distinct forms, is a mystery which must perhaps in most cases remain forever unexplained; but in some instances a satisfactory solution seems to be attainable. Sir Charles Lyell, in his Manual of Elementary Geology, describes the "Bradford apiocrinites" as having grown in an oolitic limestone, upon which their stony roots now form a continuous pavement; a layer of clay rests upon it, containing the bodies and stems of innumerable encrinites. "In this case," he remarks, "it appears that the 'Great Oolite' had supported, for a time, a thick submarine forest of these beautiful zoophytes, until the clear and still water was invaded by a current charged with mud, which threw down the stone-lilies, and broke most of their stems short off near the point of their attachment." At several localities I have observed traces of a similar succession of events. The Buttonmould Knob, in the vicinity of Louisville, affords a striking example. A thin bed of limestone, deposited near the top of the Black Slate, is made up almost exclusively of the exuviæ of encrinites, showing that these animals abounded in clear water charged with carbonate of lime. The formation changes, and the crinoids disappear, having been entombed in the argillaceous deposit in which their remains are imbedded. In places where the clay is unmixed with iron, the fossils are in an excellent state of preservation; where iron mingles with it, they are generally fragmentary and imperfect.

We have satisfied ourselves of the carnivorous habits of the crinoidea. In one of our earliest excursions, we discovered a curious encrinite with an acroculia between its fingers, as if it had been in the act of devouring it when it perished. At Cincinnati we have been shown several specimens, in which a trochus is thus entangled in the rays of *Glyptocrinus decadactylus*, and we have lately received from our friend, Mr. Worthen, of Warsaw, Ill., a platycrinus, and more than one species of actinocrinus, with an acroculia in a similar position. The gasteropods seem to have been their favorite article of food.

The prevalence of the crinoids in this country, as compared with Europe, is a fact that will strike every observer. Dr. Troost supposed that he had collected together the remains of a greater number than were at that time known to European palæontologists, and the list known to him has been already more than doubled. These organisms are to our continent what the crustaceans are to Europe—the ocean-lilies swarmed along the shores of our early seas, as the trilobites did in the ocean which has given place to dry land in the old world. Entering at the same time upon the stage of existence, at the earliest dawn of creation, the trilobites reached their meridian in the first Silurian epoch, and died out at the beginning of the Coal period. The crinoids only attained their full development in the Carboniferous era, and, extending through the Mesozoic period, have still their representatives among existing races.—*Proceedings of the American Association.*

